# Prevalence of SARS-CoV-2–Specific Antibodies, Japan, June 2020

**DOI:** 10.3201/eid2702.204088

**Published:** 2021-02

**Authors:** Takashi Yoshiyama, Yasuki Saito, Kunitsugu Masuda, Yoshiko Nakanishi, Yasutoshi Kido, Kazuhiro Uchimura, Satoshi Mitarai, Tadaki Suzuki, Yu Nakagama, Hiroshi Kubota, Maki Satomi, Sana Uchikoba, Makoto Ohnishi, Takaji Wakita, Seiya Kato, Katsunobu Kato

**Affiliations:** Research Institute of Tuberculosis, Kiyose, Japan (T. Yoshiyama, K. Uchimura, S. Miatai, S. Kato);; Fukujuji Kenshin Center of Miyagi Anti-Tuberculosis Association, Sendai, Japan (Y. Saito);; Osaka Anti-Tuberculosis Association, Osaka, Japan (K. Masuda);; Center for Comprehensive Health Check and Promotion of Japan Anti-Tuberculosis Association, Tokyo, Japan (Y. Nakanishi);; Osaka City University, Osaka (Y. Kido, Y. Nakagama);; National Institute of Infectious Diseases, Tokyo (T. Suzuki, M. Ohnishi, T. Wakita);; Osaka City University Hospital, Osaka (H. Kubota);; Ministry of Health, Labor and Welfare, Tokyo (M. Satomi, S. Uchikoba);; Government of Japan, Tokyo (K. Kato)

**Keywords:** seroprevalence, antibodies, SARS-CoV-2, COVID-19, diagnostics, immunology, antibody responses, respiratory infections, severe acute respiratory syndrome coronavirus 2, 2019 novel coronavirus disease, coronavirus disease, zoonoses, viruses, coronavirus, serology, Japan

## Abstract

We used 2 commercially available antibody tests to estimate seroprevalence of severe acute respiratory syndrome coronavirus 2 infection in Japan during June 2020. Of 7,950 samples, 8 were positive by both assays. Using 2 reliable antibody tests in conjunction is an effective method for estimating seroprevalence in low prevalence settings.

During the first wave of the coronavirus disease (COVID-19) pandemic in Japan, a total of 16,884 persons tested positive for severe acute respiratory syndrome coronavirus 2 (SARS-CoV-2) by May 31, 2020, indicating a national cumulative incidence of 0.013% (*1*,*2*) (Appendix Figure). To establish a surveillance method in low prevalence settings, we assessed the seroprevalence of SARS-CoV-2 infection in Japan in early June 2020.

## The Study

By October 2020, no standard antibody test or standardized method for estimating the seroprevalence of SARS-CoV-2 infection had been established. We used 2 serologic tests, a neutralizing antibody assay, and participant questionnaires to estimate the seroprevalence of SARS-CoV-2 infection in Japan.

We conducted a seroprevalence survey of SARS-CoV-2 infection in 3 prefectures of Japan during June 1–7, 2020. We selected 2 prefectures with a relatively high cumulative incidence of confirmed COVID-19 cases as of May 31, 2020: Tokyo, with an incidence of 0.039% (5,408 cases/13.9 million population) and Osaka, with an incidence of 0.020% (1,785 cases/8.8 million population). To better estimate the range of seroprevalence of SARS-CoV-2 infection in Japan, we also chose a prefecture with a relatively low cumulative incidence, Miyagi, with an incidence of 0.004% (88 cases/2.3 million population). 

Each prefecture was responsible for using its civil registration data to randomly select participants. The Tokyo metropolitan government used random sampling stratified by age and sex in 3 cities with a cumulative incidence resembling the average of the Tokyo metropolitan area. The Miyagi prefectural government used its residence registry to conduct random sampling with stratification for age, sex, and geographic region. The Osaka prefecture used age-adjusted random sampling to select resident users of an existing smartphone application on general health (Figure).

**Figure:**
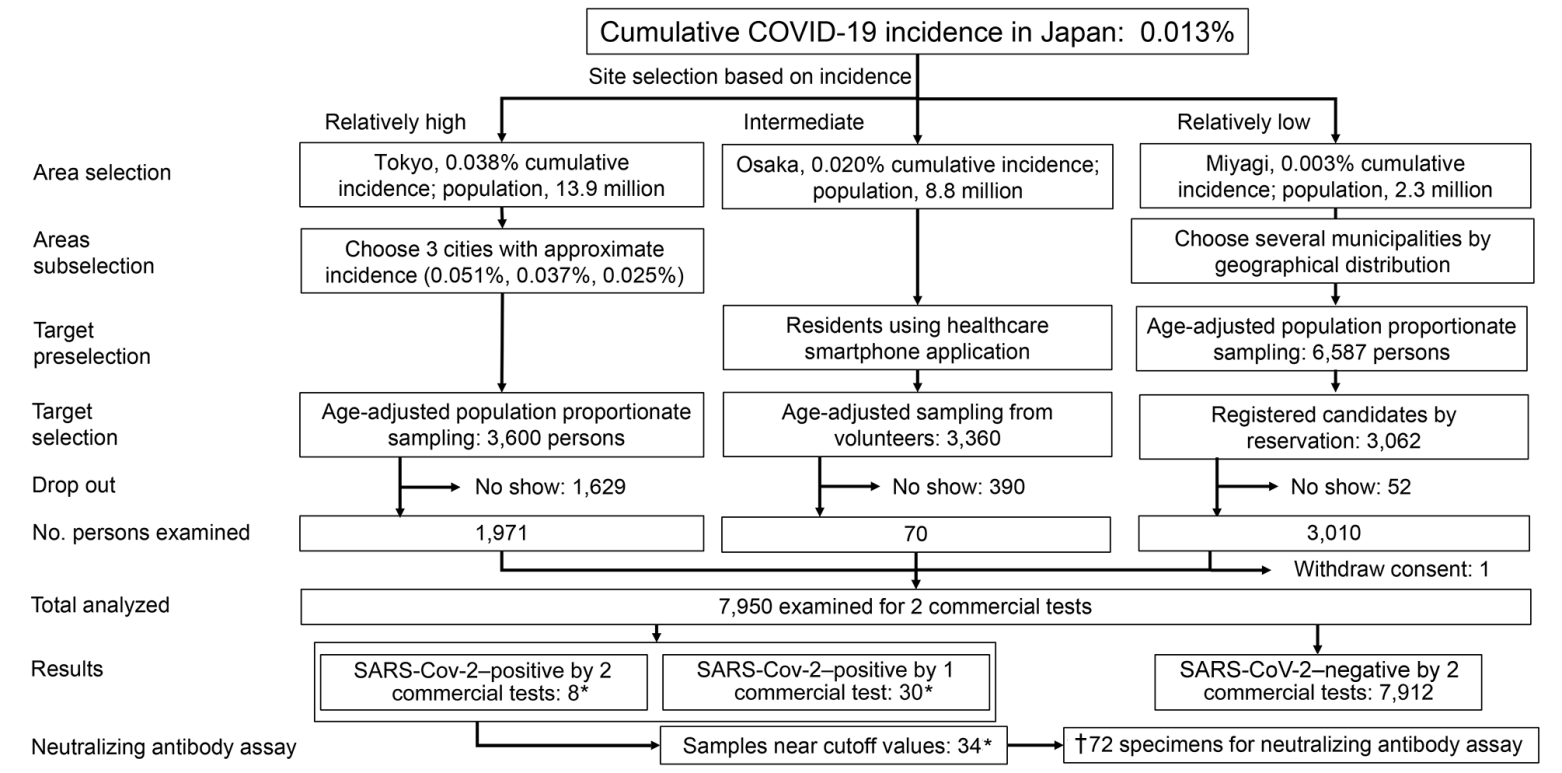
Flowchart of participants and results of SARS-CoV-2–specific antibody survey, Japan, 2020. Dagger (†) indicates sum of values marked with asterisks (*). SARS-CoV-2, severe acute respiratory syndrome coronavirus 2.

Eligible participants were persons >20 years of age living in Japan. The Tokyo and Miyagi prefectures excluded otherwise eligible participants with temperatures ≥37.5°C. All participants provided written informed consent. The study was approved by the internal review boards of the Research Institute of Tuberculosis (approval no. RIT/IRB 2020–04, 2020–05) and the National Institute of Infectious Diseases (approval no. 1140). 

First, we asked participants to complete a questionnaire (Appendix Table 1). Trained healthcare workers collected blood samples from the participants. After centrifuging the samples, the workers collected serum and tested the samples with 2 commercially available antibody tests to detect the SARS-CoV-2 nucleocapsid antigen: a chemiluminescent microparticle immunoassay with published specificity results of 99.6%–99.9% at a cutoff index of 1.4 (SARS-CoV-2 IgG assay; Abbott, https://www.abbott.com) (*3*,*4*) and an electrochemiluminescence immunoassay for the qualitative detection of antibodies with 99.8% specificity and 100% (manufacturer determined) sensitivity (Elecsys Anti-SARS-CoV-2 immunoassay; F. Hoffmann-La Roche Ltd, https://www.roche.com) (*5*). Samples that were positive or borderline negative by >1 assay (reference range 1.20–1.39 for the Abbott test and 0.70–0.99 titer for the Roche test) were sent to Japan’s National Institute of Infectious Diseases (Tokyo) for a neutralizing antibody assay with VeroE6/TMPRSS2 cells (JCRB Cell Bank accession no. JCRB1819) (*6*). For the neutralizing antibody assay, we used an in vitro cytopathic effect assay, which is more accurate than serologic tests and therefore well-suited for confirmation of results; however, only a few laboratories in Japan have the resources to conduct the assay.

We compared the 2 groups using the χ^2^ test, considering values with p<0.05 to be significant. We compared ordinal scales by using the Mann-Whitney U test. We used Excel (Microsoft, https://www.microsoft.com) to conduct statistical analyses.

In total, 13,547 persons were invited to participate in the study; 7,950 (58.7%) accepted and gave informed consent. Of the participants, 3,660 (46.0%) were men and 4,290 (54.0%) were women. Persons 20–29 years of age (877 of 1,875 invitees) or 80–99 years of age (337 of 1,102 invitees) had the lowest response rate (Appendix Table 2). Participants from Osaka were more likely to have a history of fever within the past 4 months (2.7%) than participants from Tokyo (2.2%) and Miyagi (1.2%) (Appendix Table 1).

Of the 7,950 serum samples, 8 tested positive by both tests and 30 samples tested positive by only 1 test (15 by Abbott and 15 by Roche) (Table). All 8 specimens that were positive for both commercial tests also tested positive in the neutralizing antibody assay. No other specimens, including those that tested positive or borderline negative in 1 assay, tested positive by the neutralizing antibody assay. 

**Table:** Serologic results of 2 antibody tests for severe acute respiratory syndrome coronavirus 2, Japan, June 2020*

Characteristic		Both +	Roche –, Abbott +	Roche +, Abbott –	Both –	Subtotal	% Patients positive by both tests (95% CI)
Total		8	15	15	7,912	7,950	0.10 (0.04–0.20)
Area							
Tokyo		2	2	4	1,963	1,971	0.10 (0.01–0.37)
Osaka		5	11	5	2,949	2,970	0.17 (0.05–0.39)
Miyagi		1	2	6	3,000	3,009	0.03 (0.00–0.19)
Sex							
M		3	7	5	3,643	3,658	0.08 (0.02–0.24)
F		5	8	10	4,269	4,292	0.12 (0.04–0.27)
Age, y							
20–29		3	0	0	875	878	0.34 (0.07–1.00)
30–39		3	2	1	1,210	1,216	0.25 (0.05–0.72)
40–49		0	3	7	1,589	1,599	0 (0.00–0.23)
50–59		0	2	4	1,457	1,463	0 (0.00–0.25)
60–69		1	4	0	1,315	1,320	0.08 (0.00–0.42)
70–79		1	4	1	1,128	1,134	0.09 (0.00–0.49)
>80		0	0	2	338	340	0 (0.00–1.08)
Job setting							
Working as before		4	4	3	3,091	3,102	0.13 (0.04–0.33)
Working at home		0	3	2	432	437	0 (0.00–0.84)
Working as before and at home		1	1	5	1,974	1,981	0.05 (0.00–0.28)
Not working		3	7	5	2,410	2,425	0.12 (0.03–0.36)
No information		0	0	0	5	5	0 (0.00–52.20)
Time spent outside the home, h							
0		1	4	1	1,153	1,159	0.09 (0.00–0.48)
<2		1	5	6	2,871	2,883	0.03 (0.00–0.19)
2–4		3	5	5	1,182	1,195	0.25 (0.05–0.73)
>4		3	1	3	2,704	2,711	0.11 (0.02–0.32)
No information		0	0	0	2	2	0 (0.00–84.20)
Fever at time of study							
Yes		0	0	0	16	16	0 (0.00–20.60)
No		8	15	15	7,886	7,924	0.10 (0.04–0.20)
No information		0	0	0	10	10	0 (0.00–30.90)
History of fever lasting >4 days in past 4 months							
Yes		4	1	1	155	161	2.48 (0.68–6.24)
No		4	14	14	7,756	7,788	0.05 (0.01–0.13)
No information		0	0	0	1	1	0 (0.00–97.50)
Previous PCR result							
Positive		1	0	0	0	1	100.00 (2.50–100.00)
Negative		0	0	0	33	33	0 (0.00–10.60)
Not applicable		7	15	15	7,879	7,916	0.09 (0.04–0.18)

*Roche, Elecsys Anti SARS-CoV-2 (F. Hoffmann-La Roche Ltd, https://www.roche.com); Abbott, ARCHITECT SARS-CoV-2 IgG assay (Abbott, https://www.abbott.com); +, positive; –, negative.

The proportion of participants with 2 positive test results was significantly higher among those with fever (2.5%) than those without fever (0.05%; p<0.01). The proportion of participants with 1 positive test result was not significantly different among those with fever (1.2%) and those without fever (0.36%; p = 0.25) (Appendix Table 1). These findings, validated by the neutralizing antibody assay, indicated that 2 positive test results accurately identified seropositive participants. The proportion of participants that tested positive by both tests was 0.1% in Tokyo, 0.17% in Osaka, and 0.03% in Miyagi. The ratios of seroprevalence to cumulative incidence were 2.6 in Tokyo, 8.3 in Osaka, and 8.7 in Miyagi. Seropositivity rates were highest among participants 20–39 years of age.

## Conclusions

The US Centers for Disease Control and Prevention suggests using an orthogonal testing algorithm, which considers the results of 2 independent antibody tests, in settings with low SARS-CoV-2 prevalence (*7*). Some surveys in high SARS-CoV-2 prevalence areas such as Spain (*8*), China (*9*), and Geneva, Switzerland (*10*) have not adopted this approach. We believe an orthogonal testing algorithm, such as the one used in this study, would be particularly valuable in our low prevalence setting. The 8 specimens that tested positive by both commercial antibody assays were confirmed to have neutralizing activity against SARS-CoV-2 with a neutralizing antibody assay. These results support our use of the neutralizing assay to confirm the validity of the commercial tests. Any 2 commercial tests with high sensitivity and specificity would be appropriate to use in this orthogonal testing strategy.

Our prefecture-level seroprevalence:cumulative case detection ratios (2.6–8.7) resemble those of the United States, which are ≈10 (*11*), and are lower than those of Switzerland (≈20–50) (*10*). These results indicate that Japan has monitored the pandemic as accurately as have other countries.

This study has several limitations. First, participant selection in Osaka was based on a volunteer population (i.e., users of a particular smartphone application) rather than the general community. In addition, the prefectures of Tokyo and Miyagi excluded otherwise eligible participants with temperatures ≥37.5°C. As a result, Osaka had the highest proportion of participants with fevers at the time of the survey and the highest seroprevalence. These factors might have introduced participation bias, skewing the results. Another limitation is that Tokyo had the lowest participation of participants 20–29 years of age. Because seroprevalences were higher in younger age groups, this sampling distribution might have reduced the seropositivity rate and prevalence:cumulative incidence ratio found in Tokyo. Furthermore, this study did not include participants <20 years of age. Although patients <20 years of age make up <10% of COVID-19 cases (*1*), excluding these patients might lead to an overestimation of SARS-CoV-2 infection prevalence. Finally, antibodies against SARS-CoV-2 might disappear after 60 days (*12*); however, the elapsed time might not affect levels of nucleocapsid protein antibody (*13*). Further studies on antibody levels after disease onset and recovery are essential for monitoring the course of infections.

We estimate that SARS-CoV-2 seroprevalence ranged from 0.03%–0.17% in Japan in early June 2020. Public health officials in low prevalence areas should consider using 2 antibody tests in conjunction for accurate surveillance.

Appendix Further information on prevalence of severe acute respiratory syndrome coronavirus 2–specific antibodies, Japan, June 2020.
