# Supplementary material for: Prevalence of SARS-CoV-2–Specific Antibodies, Japan, June 2020
Source: Emerg Infect Dis. 2021 Feb;27(2):628–31. doi: 10.3201/eid2702.204088 (PMC7853542; doi:10.3201/eid2702.204088)
Supplement: Appendix — Further information on prevalence of severe acute respiratory syndrome coronavirus 2–specific antibodies, Japan, June 2020. [file 20-4088-Techapp-s1.pdf]

Article DOI: https://doi.org/10.3201/eid2702.4088

Prevalence of SARS-CoV-2–Specific Antibodies, Japan, June 2020

Appendix

Sample Size Calculations

Based on an estimated 0.5% seropositive prevalence and a margin of error of 0.3%, we projected that a sample size of 2,124 participants would provide 80% power with an α error of 0.05. Each prefecture was asked to recruit 3,000 persons.

Methods for Neutralizing Antibody Assay

The cells were cultured as monolayers in Dulbecco modified Eagle medium supplemented with 5% fetal calf serum, 50 IU/mL penicillin G, and 50 µg/mL streptomycin. The SARS-CoV-2 strain 2019-nCoVJPN/TY/WK-521/2020 (GISAID ID: EPI_ISL_408667), originally isolated with VeroE6/TMPRSS2 cells from a COVID-19 infected patient, was used as the challenge virus. Serial 2-fold dilution of the test serum (from 1:5 to 1:320) and equal amounts of the prepared challenge virus (100 units of 50% tissue culture infectious dose) solution were mixed at 37°C for 1 hour, followed by the addition of 100 µL of VeroE6/TMPRSS2 cells (10^4 cells).

After 5 days of incubation at 37°C, the presence or absence of a cytopathic effect in each well was observed by using an inverted microscope. After formalin fixation, the cells were stained with a crystal violet solution for the final evaluation.

Appendix Table 1. Characteristics of participants in serologic survey for severe acute respiratory coronavirus 2 infection, Japan, June 2020

	Prefecture			
Characteristics	Tokyo	Osaka	Miyagi	Subtotal
Total	1,971	3,009	2,970	7,950
Work setting				
Working outside of home	510	1,181	1,411	3,102
Working at home	192	149	96	437
Working both outside and at home	685	820	476	1,981
Not working	580	819	1,026	2,425
No information	4	1	0	5
Time spent outside the home each day, h				
0	352	385	422	1,159
<2	724	986	1,173	2,883
2–4	319	504	372	1,195
>4	574	1,095	1,042	2,711
No information	2	0	0	2
Fever at time of study				
Yes	1	4	11	16
No	1,960	2,966	2,998	7,924
No information	10	0	0	10
History of fever lasting >4 days in past 4 months				
Yes	43	82	36	161
No	1,927	2,888	2,973	7,788
No information	1	0	0	1
Previous PCR result				
Positive	1	0	0	0
Negative	11	17	5	33
Not applicable	1,959	2,953	3,004	7,916

Appendix Table 2. Participants in serologic survey for severe acute respiratory coronavirus 2 infection, Japan, June 2020

Location,	M			F			Total		
age range	Invited	Participated	%	Invited	Participated	%	Invited	Participated	%
Tokyo									
20–29	292	92	31. 5	299	132	44. 1	591	224	37. 9
30–39	298	127	42. 6	301	201	66. 8	599	328	54. 8
40–49	323	172	53. 3	311	217	69. 8	634	389	61. 4
50–59	288	177	61. 5	275	196	71. 3	563	373	66. 3
60–69	251	157	62. 5	250	170	68. 0	501	327	65. 3
70–79	194	103	53. 1	201	121	60. 2	395	224	56. 7
≥80	145	54	37. 2	172	52	30. 2	317	106	33. 4
Total	1,791	882	49. 2	1,809	1,089	60. 2	3,600	1,971	54. 8
Osaka									
20–29	237	194	81. 9	249	210	84. 3	486	404	83. 1
30–39	248	219	88. 3	261	235	90. 0	509	454	89. 2
40–49	325	289	88. 9	340	308	90. 6	665	597	89. 8
50–59	287	259	90. 2	301	282	93. 7	588	541	92. 0
60–69	235	208	88. 5	246	226	91. 9	481	434	90. 2
70–79	250	214	85. 6	263	228	86. 7	513	442	86. 2
≥80	50	42	84. 0	68	56	82. 4	118	98	83. 1
Total	1,632	1,425	87. 3	1,728	1,545	89. 4	3,360	2,970	88. 4
Miyagi									
20–29	408	103	25. 2	390	146	37. 4	798	249	31. 2
30–39	500	192	38. 4	494	243	49. 2	994	435	43. 8
40–49	599	284	47. 4	579	330	57. 0	1,178	614	52. 1
50–59	502	238	47. 4	502	308	61. 4	1,004	546	54. 4
60–69	508	248	48. 8	530	314	59. 2	1,038	562	54. 1
70–79	423	226	53. 4	485	244	50. 3	908	470	51. 8
≥80	244	62	25. 4	423	71	16. 8	667	133	19. 9
Total	3,184	1,353	42. 5	3,403	1,656	48. 7	6,587	3,009	45. 7

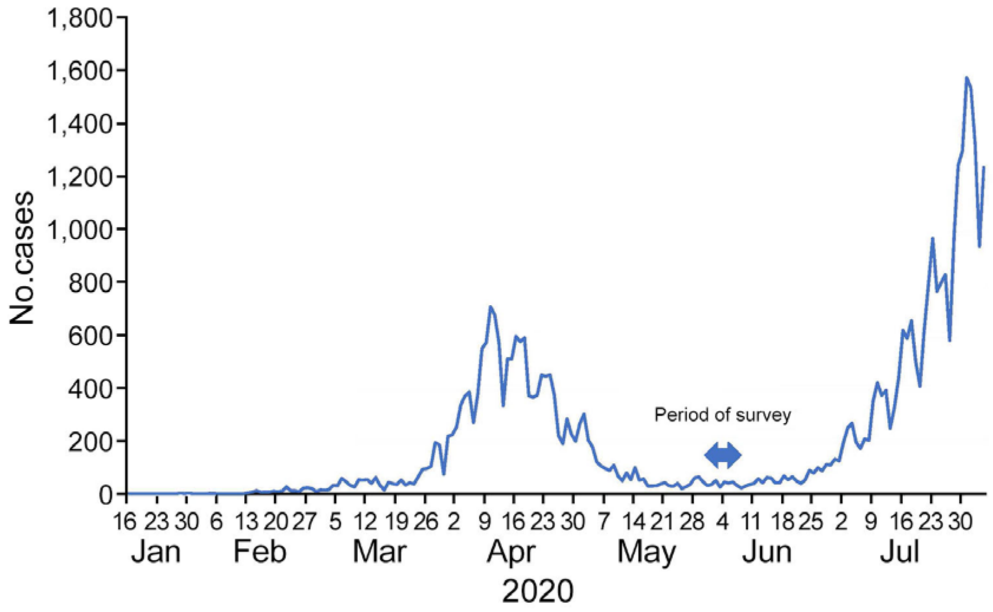

Appendix Figure 1. Case detection of coronavirus disease, Japan, June 2020.
